# The Impact of Lesser Trochanter Displacement on Hip Flexor Strength Recovery in Patients With Trochanteric Fracture

**DOI:** 10.7759/cureus.73095

**Published:** 2024-11-05

**Authors:** Takato Nishida, Yoshiki Fujikawa, Yuki Nagamune

**Affiliations:** 1 Division of Physical Therapy, Faculty of Rehabilitation and Care, Seijoh University, Aichi, JPN; 2 Department of Rehabilitation, Aichi-Pref Saiseikai Rehabilitation Hospital, Aichi, JPN

**Keywords:** hip flexion strength, lesser trochanter displacement, repeated measures anova, trochanteric fracture, walking ability

## Abstract

Introduction: Trochanteric fractures (TFs) are common in older individuals and are expected to increase with Japan's aging population. These fractures often result in poor long-term outcomes, such as decreased independent walking and reduced hospital discharge rates. A significant aspect of TF involves displacement of the lesser trochanter (LT), which can weaken hip flexor muscles and potentially affect the recovery of activities of daily living (ADLs), including walking. Previous research has shown conflicting results regarding the effect of lesser trochanteric displacement on hip function and walking ability. This study aimed to determine whether displacement of the LT affects the recovery of hip flexor strength and walking ability at discharge in patients with TF.

Methods: This prospective cohort study included 29 patients with TF admitted to a rehabilitation hospital between April 2023 and June 2024. The patients were classified into two groups: the LT displacement and the non-LT (NLT) displacement groups. Muscle strength (hip flexion, abduction, and knee extension) was measured using a handheld dynamometer. Walking ability assessments included gait speed, timed up-and-go test (TUG), 6-minute walk test (6 MWT), and functional ambulation category (FAC). Cognitive function was evaluated using the Hasegawa Dementia Rating Scale-Revised (HDS-R). Statistical analyses included repeated-measures analysis of variance (ANOVA) for muscle strength comparisons over time, with adjustments for violations of sphericity using the Greenhouse-Geisser correction.

Results: There were no significant differences between the LT and NLT groups in terms of demographic characteristics such as age, sex, or cognitive function. Repeated-measures ANOVA revealed a significant difference in hip flexor strength on the injured side between the groups, with the LT group showing persistent weakness until discharge. Significant improvements were noted in hip abduction and knee extension strength on the injured side, although no group differences were observed. Post-hoc analysis indicated significant strength improvements over time, particularly between admission and discharge, for most muscle groups, except for hip flexor strength in the LT group.

Conclusion: Lesser trochanteric displacement in patients with TF resulted in a specific decline in hip flexor strength on the injured side, which persisted until discharge. However, no significant impact on walking ability was observed, likely because of compensatory mechanisms involving other muscles.

## Introduction

Trochanteric fractures (TFs) are the most common type of proximal femur fractures in the older population [1,2]. In Japan, a country that has become a super-aged society, the incidence of TF is expected to increase with population aging [3]. Furthermore, TF is associated with poor long-term outcomes, such as low rates of independent walking and reduced rates of hospital discharge [4,5]. Given the high frequency of TF and its significant adverse impact on patients' post-injury lives, it is essential to aim for functional recovery through rehabilitation and help patients regain their pre-injury lifestyles.

A characteristic feature of TF is that the lesser trochanter (LT) is displaced in 60.7% of patients because of femoral shaft fractures [6]. The LT remains missing from the femur after surgery, as it was not fixed during the procedure. Since the hip flexor muscles attach to the LT, displacement may result in reduced hip flexor strength, which could potentially hinder the recovery of activities of daily living (ADLs), including walking.

Previous studies on patients with displaced LTs have reported that such displacement impairs the ability to regain independent walking and contributes to persistent pain [7,8]. However, other reports suggest that there are no significant differences in hip function impairment based on the presence or absence of lesser trochanteric displacement, and no clinical disadvantages have been observed [9,10]. Additionally, most studies comparing hip flexor strength have been cross-sectional, with few investigating the longitudinal course of hip flexor strength recovery [11]. Thus, it remains unclear whether LT displacement impedes the recovery of hip flexor strength and adversely affects walking ability.

Clarifying the recovery process of hip flexor strength following LT may provide valuable insights into adjusting the intensity and frequency of strength training during postoperative rehabilitation. Therefore, this study aimed to determine the impact of lesser trochanteric displacement on the recovery of hip flexor strength and walking ability at discharge in patients with TF.

## Materials and methods

Participants

Measurements were conducted between April 2023 and June 2024. The participants were individuals who had a TF and were hospitalized at Aichi-Pref Saiseikai Rehabilitation Hospital during this period. The exclusion criteria were the inability to give consent to participate in the study, inability to understand instructions during the measurements, motor paralysis of the lower limbs or trunk, a history of proximal femur fracture on the same side as the fracture side, and incomplete or untraceable measurement data.

The study was conducted in accordance with the Declaration of Helsinki, and written informed consent was obtained from each participant prior to participation. This study was approved by the Ethics Committee of Aichi-Pref Saiseikai Rehabilitation Hospital (approval no. 2022201). The first author takes full responsibility for the integrity and accuracy of the data analysis.

Measures

In this prospective cohort study, participant data included variables such as age, gender, time elapsed since injury, acute phase duration, weight, cognitive status, muscle strength, walking ability, and presence or absence of LT. Muscle strength was measured during hip flexion, abduction, and knee extension on both sides. Maximal isometric muscle strength was assessed with a handheld dynamometer (μTasF-1; Anima Corporation, Tokyo, Japan). The measurement method used a fixation belt, which has been reported to have high intra- and inter-examiner reliability in previous studies [12,13]. Each measurement was performed twice, and the maximum value was used for analysis. A 60-second rest period was provided between measurements. Hip flexor strength was measured in the sitting position, with the hip and knee joints at 90° of flexion. The sensor pad was placed on the distal anterior thigh, and the examiner secured the fixation belt. Hip abduction strength was measured in the supine position, with the hip in a neutral position. The sensor pad was placed on the distal lateral thigh, and the fixation belt was fixed to the contralateral distal thigh. Knee extensor strength was measured in the sitting position with the knee joint at 90°. The sensor pad was placed on the distal anterior aspect of the lower leg, and the belt was fixed to a post behind the lower leg. The torque-to-body weight ratio (Nm/kg) was calculated by multiplying the representative strength value (N) by leg length (m) at the measurement site and dividing by body weight (kg). This ratio was used for analysis.

Walking ability was assessed using gait speed, timed up-and-go test (TUG), 6-minute Walk Test (6 MWT), and functional ambulation category (FAC). For gait speed, participants walked 16 m at a comfortable pace, with 3-m zones at each end and a 10-m zone in the middle. The time taken to pass through the middle 10 m was recorded to calculate gait speed (m/s). In the TUG test, participants were instructed to stand up from a chair, walk 3 m, turn, walk back, and sit down as quickly as possible [14]. For the 6 MWT, participants walked for 6 min at a comfortable pace, and the total distance walked (m) was recorded. A cane or walker was allowed if necessary [15]. The FAC was used to assess the maximum walking ability of the participants, with scores ranging from 0 to 5, with higher scores indicating greater independence [16].

Cognitive status was assessed using the Hasegawa Dementia Rating Scale-Revised (HDS-R) [17], which is widely used in Japan to screen for cognitive function. The HDS-R scores range from 0 to 30, with lower scores indicating poorer cognitive function.

The presence or absence of lesser trochanteric displacement was determined based on previous studies [18]. Participants with a displacement > 2 mm, as observed in x-ray and CT scan images, were classified into two groups: the LT and the non-LT (NLT) displacement groups.

Statistical analysis

The normality of the data was confirmed for each group and variable using the Shapiro-Wilk test. Variables with a normal distribution were tested for homogeneity of variance using the F-test, and unpaired t-tests were performed. Fisher’s exact test was used to analyze nominal data. To compare muscle strength over time between the NLT and LT groups, torque-to-body weight ratios for hip flexion, abduction, and knee extension were analyzed using repeated-measures analysis of variance (ANOVA). This was performed with between-group and time factors at three points: admission, one-month post-admission, and discharge. Mauchly's Test of Sphericity was used to confirm the assumption of equal variance. When sphericity was violated, Greenhouse-Geisser corrected values were applied. For variables that showed significant differences at the three time points, post hoc tests were conducted using the Bonferroni method.

All statistical analyses were conducted using EZR ver.1.53 (Jichi Medical University, Saitama, Japan), a modified version of R Commander, designed to include statistical functions commonly used in biostatistics [19]. The significance level was set at 5%.

## Results

**Figure 1:**
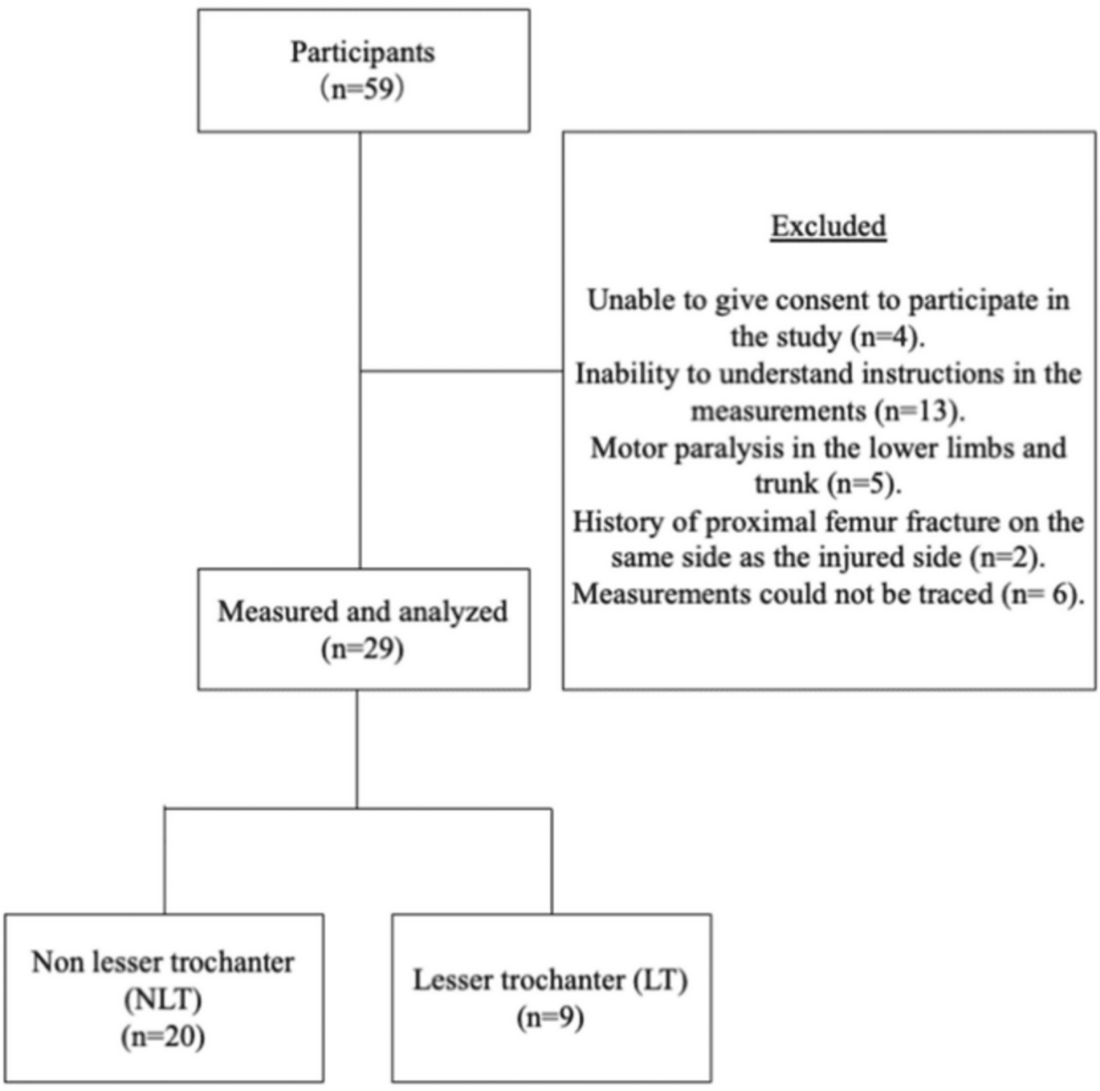
Flowchart of the selection process of the study participants. LT, lesser trochanter displacement group; NLT, non-lesser trochanter displacement group

The final analysis included data from 29 participants (Figure 1). Table 1 shows the results of group comparisons of demographic characteristics and walking ability at discharge. No significant differences were observed between the groups in terms of age, sex, elapsed days, acute-phase days, HDS-R scores, gait speed, TUG test result, 6 MWT result, or FAC scores.

**Table 1:** Comparison of demographic characteristics and walking ability at discharge LT: Lesser trochanter group; NLT: Non-lesser trochanter group; Elapsed days: The number of days from the date of sustaining a trochanteric fracture to discharge from Aichi-Pref Saiseikai Rehabilitation Hospital.; Acute phase days: The number of days from the date of sustaining a trochanteric fracture to admission to Aichi-Pref Saiseikai Rehabilitation Hospital.; HDS-R: Hasegawa Dementia Rating Scale-Revised; TUG: Timed Up and Go test; 6 MWT: 6-minute Walk Test; FAC: Functional Ambulation Category; *: Unpaired t-test; †: Fisher's exact test

		LT (n = 9)	NLT (n = 20)	P-value
Age*		85.2 ± 5.0	83.6 ± 8.9	0.6
Gender ^†^	Male	1	6	0.38
	Female	8	14	
Elapsed days*		82.4 ± 19.3	81.3 ± 17.1	0.87
Acute phase days*		15.0 ± 6.4	21.1 ± 8.4	0.06
HDS-R*		21.7 ± 7.2	24.3 ± 4.4	0.23
Gait speed (m/s)*		0.6 ± 0.3	0.8 ± 0.4	0.17
TUG (s)*		23.8 ± 16.0	18.3 ± 8.2	0.30
6 MWT (m)*		274.8 ± 77.4	274.5 ± 119.2	0.99
FAC ^†^	0	0	0	0.83
	1	0	0	
	2	1	2	
	3	2	3	
	4	2	8	
	5	4	7	

The results of the repeated measures ANOVA are presented in Table 2. Based on the results of the Shapiro-Wilk test, the torque-to-body weight ratios for each time point and group followed a normal distribution. Additionally, Mauchly’s Test of Sphericity revealed that sphericity was violated for knee extension on the injured side; therefore, Greenhouse-Geisser-corrected values were used. Repeated-measures analysis ANOVA indicated a significant main effect between the groups only for hip flexion on the injured side (p < 0.01). For hip abduction and knee extension on the injured side, as well as knee extension on the non-injured side, significant main effects were observed based on the time factor (hip abduction on the injured side, p < 0.01; knee extension on the injured side, p < 0.01; knee extension on the non-injured side, p = 0.01). No interactions were detected for any of the variables. Furthermore, a post hoc analysis using the Bonferroni method was conducted for variables that showed significant differences in the repeated-measures ANOVA. Although hip flexion on the injured side demonstrated overall significance, no significant differences were observed between specific time points. For hip abduction and knee extension on the injured side, significant differences were found between measurements at admission and those taken one month later, as well as between measurements at admission and those at discharge (p < 0.05). Knee extension on the non-injured side showed a significant difference between admission and one month later only (p < 0.05).

**Table 2:** Repeated-measures ANOVA and post hoc test of torque-to-body weight ratios across measurement sites Interaction: This indicates differences in effects between groups at different measurement points, showing that the effect of one factor (e.g., measurement time point or group attribute) changes depending on another factor. When the interaction effect is significant, the differences between groups at each time point are statistically distinct.; Post hoc test: For repeated-measures ANOVA, when a significant difference was found within groups, post hoc tests using the Bonferroni method were conducted to determine between which groups the differences existed.

		Between-group	Within-group	Interaction	Post hoc test
Hip flexion	Fracture side	0.01	0.05	0.91	No significant
	Non-fracture side	0.17	0.72	0.79	Admission < 1 month later, discharge
Hip abduction	Fracture side	0.21	< 0.01	0.98	-
	Non-fracture side	0.36	0.14	0.51	-
Knee extension	Fracture side	0.43	< 0.01	0.63	Admission < 1 month later, discharge
	Non-fracture side	0.78	0.01	0.77	Admission < 1 month later

## Discussion

This study conducted a longitudinal investigation to determine whether lesser trochanteric displacement in patients with TF affects the recovery of hip flexor strength. The results showed that patients with lesser trochanteric displacement had weaker hip flexor strength on the fracture side, which persisted until discharge. No differences in strength were observed for the other muscles based on lesser trochanteric displacement, and muscle strength tended to improve from admission to discharge. These findings suggest that LT may result in a specific decline in hip flexor strength and hinder subsequent recovery.

One potential factor for muscle weakness could be pain following hip fractures, as pain is known to negatively affect muscle strength output [20]. Chronic pain can lead to muscle atrophy and reduced muscle strength [21]. However, none of the participants reported pain during the measurements, and significant improvements in hip abductor and knee extensor strength were observed from admission to discharge. Therefore, pain is unlikely to be the main factor responsible for the decline in hip flexor strength. LT may have induced specific changes, such as shortening or atrophy, in the hip flexor muscles, potentially resulting in reduced muscle strength. Previous studies have reported that displacement of the LT leads to atrophy of the iliopsoas muscle and fat infiltration [22]. When muscle atrophy occurs alongside the replacement of muscle fibers with fat tissue, the cross-sectional area of the muscle decreases, reducing the amount of contractile tissue and subsequently leading to muscle weakness. Another potential factor is the effect of displacement on muscle length and tension. According to the length-tension relationship curve, muscle shortening may result in an inability to generate maximum tension. Cross-sectional studies, including that by Aprato et al., have reported that displacement of the LT impairs iliopsoas muscle function, leading to reduced hip flexor strength [11,18]. Additionally, the length of the iliopsoas muscle plays an important role in walking, and it has been shown that it is more affected by changes in length than other muscles, such as the biceps femoris and semitendinosus [23]. Based on these findings, the group with LT experienced a prolonged inability to generate maximum tension in the iliopsoas muscle, resulting in differences in hip flexor strength recovery.

The influence of muscle weakness on walking ability suggests that shortening the iliopsoas muscle may negatively affect walking performance [11]. However, despite a significant decrease in hip flexor strength on the fracture side in the lesser trochanteric displacement group, no significant decline in walking ability was observed. Hip flexion during walking primarily occurs during the swing phase, and this movement involves not only the iliopsoas muscle but also muscles such as the rectus femoris and sartorius [24]. These muscles may compensate for the weakness of the iliopsoas muscle, allowing participants to maintain their walking ability. Compensation may be more pronounced in cases where muscle weakness is partial or when walking aids are used. Thus, even though there was a decline in iliopsoas muscle strength, other muscles may have compensated, preventing a significant impact on walking speed or walking ability indicators, such as the TUG test result. Second, differences in walking patterns may have influenced the iliopsoas muscle activity. It has been reported that the muscle activity required during walking varies depending on the walking pattern [25]. For example, patients using a walker experience reduced weight-bearing and anterior pelvic tilt, which decreases the muscle activity required to swing the leg forward. In this study, similar to previous studies, it is possible that walker users required less iliopsoas muscle activity than independent walkers, which may explain why the weakness of the iliopsoas muscle did not significantly affect walking ability. In summary, despite the reduction in hip flexor strength, compensatory activity from other hip flexor muscles and reduced load associated with different walking patterns may have contributed to the maintenance of walking ability.

This study had some limitations. First, muscle activity during walking was not measured. Although this study assessed iliopsoas muscle weakness, the activities of the iliopsoas and other hip flexor muscles during walking were not directly measured. Thus, it cannot be conclusively stated that other muscles compensate for the weakness of the iliopsoas muscle without conducting a muscle activity analysis such as electromyography (EMG). Secondly, the influence of different walking patterns was not fully considered. Although this study included patients using walking aids, the burden on the iliopsoas and other muscle groups may differ depending on the walking pattern. Subgroup analyses based on conditions such as independent walking or walker use are necessary to examine the impact of walking patterns in detail. Third, the sample size was small, limiting the generalizability of the results regarding compensatory mechanisms of specific muscle groups or differences in walking patterns. Therefore, future studies with larger sample sizes are warranted. Finally, the degree of displacement of the LT was not evaluated. It is possible that iliopsoas muscle function was further restricted depending on the degree of displacement. Considering these limitations, future research should focus on measuring actual muscle activity using EMG, performing detailed analyses of different walking patterns, assessing the degree of LT, and expanding the sample size to gain a more precise understanding of the relationship between hip flexor weakness and walking ability.

## Conclusions

This study revealed that in patients with TF, displacement of the LT was specifically associated with a reduction in hip flexor strength on the fracture side, which persisted until discharge. These findings suggest that displacement of the LT may lead to the shortening of the iliopsoas muscle or the replacement of muscle fibers with adipose tissue, hindering muscle strength recovery. Furthermore, despite the decrease in hip flexor strength, no significant effect on walking ability was observed. This could be attributed to the possibility that other muscle groups compensate for the weakness of the iliopsoas, thereby mitigating the effects of muscle strength deficiencies.
